# Neonatal and pediatric thymic grafts generate similar human T-cell chimerism in humanized mice

**DOI:** 10.3389/fimmu.2026.1852823

**Published:** 2026-07-06

**Authors:** Natalia M. Del Rio, Liupei Huang, Alexis M. Holm, Wesley Blashka, Nicholas A. Smith, Sayandeep Saha, Adam J. Bell, Janell Richardson, Katherine E. Badior, Christian J. Kastrup, Matthew E. Brown

**Affiliations:** 1 Department of Surgery, School of Medicine and Public Health, University of Wisconsin-Madison, Madison, WI, United States; 2 Medical College of Wisconsin, Milwaukee, WI, United States; 3 Taconic Biosciences, Inc., Rensselaer, NY, United States; 4 Versiti Blood Research Institute, Milwaukee, WI, United States; 5 Department of Surgery, Division of Trauma and Acute Care Surgery, Medical College of Wisconsin, Milwaukee, WI, United States; 6 Departments of Biochemistry, Biomedical Engineering, and Pharmacology and Toxicology, Medical College of Wisconsin, Milwaukee, WI, United States

**Keywords:** BLT mouse, humanized mouse, mRNA vaccine, NeoThy mouse, thymopoiesis, thymus transplantation

## Abstract

Human immune system (HIS) humanized mice, particularly those incorporating human thymic tissue and hematopoietic stem/progenitor cell (HSPC) co-transplantation, provide a powerful *in vivo* platform to study human T-cell development, tolerance, and effector function in the context of a full human immune system. The thymus is a lymphoid organ that is indispensable for generating a diverse and self-tolerant T-cell repertoire, and its inclusion in HIS models is essential for generating human major histocompatibility complex-restricted T cells. The bone marrow–liver–thymus (BLT) HIS model is made using human fetal tissues of a very narrow gestational age, typically 17–22 weeks. We previously developed the NeoThy™ humanized immune system mouse model, which is created using cord blood HSPCs and donated thymus tissue from neonatal and pediatric donors, as a non-fetal alternative to the BLT model. While the BLT model uses tissue of relatively consistent gestational age, further study is needed to assess the impact of the thymic donor age in the NeoThy model, since thymic tissue donors can range from a few days old to many years old. Here, we created multiple cohorts of NeoThy mice using thymic tissue from donors of various ages and compared them to each other and to cohorts of BLT mice. In the NeoThy mouse cohorts, we confirmed *de novo* thymopoiesis within transplanted human thymic grafts. Across multiple independent experiments encompassing both neonatal and pediatric thymic donors, we observed similar T-cell chimerism and function, regardless of thymic donor age. Inter-donor variability, an inherent feature of all HIS mouse models, accounted for observed differences in immune engraftment kinetics. T cells developing in the presence of a transplanted thymus exhibited gene expression signatures consistent with more mature functional responses to mRNA vaccine challenge than those observed in mice receiving cord blood only. These findings highlight the importance of including thymic transplantation in this context and confirm that both neonatal and pediatric thymus donor tissue are capable of generating robust chimeric immune systems in HIS mice.

## Highlights

- Inter-donor variability, not donor age, is the primary influencer of T-cell reconstitution across fetal, neonatal, and pediatric thymic grafts.
- Fetal BLT mice have higher, supraphysiologic frequencies of T cells at later time points post-humanization.
- NeoThy mouse T-cell gene expression signatures are consistent with greater functional maturation in the context of mRNA virus challenge than cord blood-only HIS mice.

## Introduction

The thymus is a primary lymphoid organ essential for the generation of naïve T cells and the establishment of central tolerance (1). Within the thymic microenvironment, hematopoietic T-cell precursors undergo T-cell receptor (TCR) rearrangement and positive and negative selection, processes critical for self-tolerance and immune competence. However, after a period of robust fetal and postnatal growth, starting in adolescence, the thymus undergoes progressive involution with age, characterized by reduced thymopoiesis, diminished epithelial cell function, and adipose replacement, ultimately contributing to immunosenescence and increased susceptibility to infection and cancer in older individuals (2).

Human immune system (HIS) humanized mice are created by engrafting immunodeficient mice with human blood (3) or hematopoietic stem/progenitor cells (HSPCs), often in combination with transplantation of fetal (4, 5) or neonatal (6, 7) human thymic tissue, enabling *in vivo* study of human immune development, tolerance, disease, and functional capabilities. These models have been instrumental in investigating questions related to transplantation and regenerative medicine (8), infectious diseases (e.g., EBV (4) and HIV (9)), toxicology (10), and immuno-oncology (11, 12). A widely used HIS model, the bone marrow–liver–thymus (BLT) mouse, relies on human fetal tissue (typically 17–22 gestational weeks) as an HSPC and thymus donor source (4, 13). While the BLT model has been invaluable for a wide range of research applications, its reliance on fetal material raises ethical and logistical constraints that restrict access (14). Limited tissue quantities also impact its scalability (14). Furthermore, while there are clear use-case advantages for these fetal tissue-based models (e.g., Zika virus (15) or other infectious diseases known to result in fetal infection and/or loss), the tolerogenic nature of the fetal immune system may be suboptimal for modeling postnatal allograft rejection (16), tumor-immune interactions, and other immune responses of children and adults (12, 17).

The NeoThy HIS model was developed to experimentally interrogate the more mature immune systems of children and adults relative to the fetus (17–19). In NeoThy mice, co-transplantation of human neonatal thymus tissue under the renal capsule, coupled with intravenous injection of cord blood-derived HSPCs, enables robust, *de novo* human thymopoiesis and multilineage immune reconstitution. The NeoThy model has since been validated across multiple institutions (7, 20, 21) and applications, including HIV infection (20, 21), regenerative therapy studies (22), and multiple other ongoing studies (e.g., immuno-oncology), highlighting its reproducibility and burgeoning potential as a translational model.

Although incorporation of human thymic grafts offers clear advantages in HIS models, T cells can still arise in animals lacking these tissues (i.e., mice receiving human cord blood alone) via education by the murine thymus and/or through extrathymic development (23–25), underscoring the need to more precisely define the contribution of the transplanted thymus tissue. Furthermore, the availability of non-fetal thymus tissue from prepubescent donors (i.e., both neonatal and pediatric patients) for HIS mice has prompted questions regarding whether thymic donor age affects thymopoiesis or immune reconstitution compared with the demonstrated high frequency of T cells within the relatively “younger” BLT model. While thymic donor age is fixed in BLT mice and therefore renders studies of donor-age variation irrelevant in that model, the wide range of possible neonatal and pediatric thymus donors for the NeoThy model opens a new area of research regarding thymus age-associated effects on T-cell development. A 2023 report by Talaie et al. (25) examined small cohorts of four or five humanized mice generated with neonatal thymus donors of different ages and reported a failure or partial failure of two cohorts (from a 2-month-old donor and a 4-month-old donor). The authors suggested that younger-aged donor thymic tissue (~2 weeks of age) might result in improved T-cell reconstitution compared with older donors (>2 months old). While this study, utilizing a modified iteration of the NeoThy model, contributed to the HIS literature, it was limited by small sample sizes and likely substantial inter-donor variability, making it difficult to draw definitive and broadly applicable conclusions. Additionally, this study and others (21, 26) illustrate the importance of standardization of HIS model creation protocols (i.e., when different sites use different methods to create ostensibly similar models, it can be difficult to compare and reproduce findings).

Our research team has developed a bank of cryopreserved and HLA-typed neonatal and pediatric thymus tissue from dozens of prepubescent donors. This resource provides an unprecedented opportunity to rigorously evaluate and reproduce donor-age effects using cohorts (i.e., batches of humanized mice created in one surgical session) comprising multiple donors. In this study, we systematically evaluated HIS mice generated from fetal tissue of approximately 16–18 weeks of gestation and from a neonatal and pediatric thymic donor tissue set spanning from 3 days to more than 2 years of age to test the impact of thymic donor age on *de novo* thymopoiesis, T-cell chimerism, and functional capacity. Our findings show that neonatal and pediatric thymic donor age has a negligible impact on human T-cell and other immune reconstitution, whereas donor-to-donor variability remains a key determinant of immune output and variability among individual mice and across experiments. Our results will help to inform the design and interpretation of future humanized mouse studies investigating the biology of thymic aging prior to involution (2, 27–29) initiation during puberty, in addition to the development of donor-specific clinical interventions to replace or rejuvenate thymic function in pathological settings such as HIV or DiGeorge syndrome (30–32).

## Materials and methods

### Animals and tissues

The human tissue samples used in this study consisted of umbilical cord blood (UCB)- derived HSPCs and neonatal thymus tissue collected under informed consent and subject to human ethics approval by the relevant institutional review boards (IRBs). Neonatal and pediatric thymus tissue was sourced from discarded medical waste tissue obtained from male and female cardiac surgery patients at the University of Wisconsin Hospital and Clinics’ American Family Children’s Hospital. HLA typing was performed on samples using the LinkSeq method, as previously described (7), and is noted in the text for the given cohorts. Human UCB or UCB-derived HSPCs were obtained from consenting patients at UnityPoint Health-Meriter Hospital in Madison, WI, or from a commercial vendor (CGT Global, Sacramento, CA). Cryopreserved human fetal tissue previously collected for projects conducted before 2019 was obtained with IRB-approved informed consent through Advanced Bioscience Resources (Alameda, CA). No new fetal tissue was obtained for this project; only existing stocks were used. The research presented in this manuscript was approved by the University of Wisconsin-Madison Health Sciences IRB and complied with federal and state laws.

Animals were housed in specific pathogen-free environments with approval from the University of Wisconsin-Madison Institutional Animal Care and Use Committee (IACUC). Humanized mice were generated using 6–8-week-old immunocompromised host animals, as previously described in Del Rio et al. (17) The base strains of immunodeficient mice used were NOD.Cg-Prkdc^scid^ Il2rg^tm1Wjl^ (NSG) (The Jackson Laboratory, cat. no. 00557), NOD.Cg-^KitW-41J^ Tyr^+^ Prkdc^scid^ Il2rg^tm1Wjl^/ThomJ (NBSGW) (33) (The Jackson Laboratory, cat. no. 026622), and NOD.Cg-Prkdc^scid^ Il2rg^tm1Sug^Tg(CMV-IL6)1-1Jic/JicTac (NOG-IL6) (Taconic Biosciences, cat. no. 13686). UCB and neonatal thymus donor tissues were matched on at least one HLA class I (A, B, or C) allele and at least one HLA class II (DRB1) allele, i.e., a partial match, except where noted (e.g., in total mismatch pairings where there are no common HLA alleles at class I or class II). Thymus donor age groups were defined as follows: young neonatal (0 to <4 months of age), intermediate (4 to <7 months), and older pediatric (>7 months to 12 years). An anti-CD2 antibody solution was used to deplete passenger thymocytes by diluting sterile antibody to a concentration of 2 mg/mL in Hank’s Balanced Salt Solution (HBSS), with 100 µg of antibody in a volume of 50 μL administered to each mouse in two doses (Day 0 and Day 7 post-surgery). A busulfan injection of 20 mg/kg was used as a myeloablative procedure in all models except the NBSGW to allow for the engraftment of human cells into the mouse bone marrow. Two 1 mm x 1 mm thymus fragments were surgically inserted between the capsule and the kidney during the humanization surgery under isoflurane anesthesia (induction: vapor setting 2.5%–4%, oxygen flow 60 mL/kg/min; maintenance: vapor setting 1.5%–3%, oxygen flow 20 mL/kg/min). Mice were given Baytril antibiotics for 10 days.

### Vaccination with SARS-CoV-2 spike protein mRNA

Research-grade vaccines were custom produced to mimic the Moderna SARS-CoV-2-spike-encoding mRNA vaccine mRNA-1273 using publicly available sequence information and a CleanCap cleanup and N1mUTP substitution approach (34). mRNA was dissolved in sodium acetate, pH 4, and combined with a lipid mixture of ALC-0315, distearoylphosphatidylcholine (Avanti Polar Lipids, Alabaster, AL, USA), cholesterol Sigma Aldrich (Burlington, MA, USA), and 1,2-dimyristoyl-rac-glycero-3-methoxypolyethylene glycol-2000 (Avanti Polar Lipids) at a ratio of 50:10:38.5:1.5% (mol/mol), respectively, using an amine-to-phosphate ratio of 6. mRNA lipid nanoparticles were dialyzed 1,000-fold against Dulbecco’s phosphate buffered saline lacking calcium and magnesium (DPBS, pH 7.4), and encapsulation was quantified using a RiboGreen assay (Quant-IT RiboGreen RNA Assay Kit, ThermoScientific (Waltham, MA, USA)). LNP composition and dosing were performed to a concentration of 0.1mg/mL, with animals receiving 5 µg mRNA in 50 µL PBS by intramuscular injection at 24 weeks post-humanization. Animals received a booster dose six weeks later (30 weeks post-humanization), and studies were terminated one week after the second dose (31 weeks post-humanization).

### Flow cytometry

Combinations of the following antibodies were used in flow cytometry to detect specific human markers: mouse anti-human CD45 (clone HIB30, BD Biosciences, cat. no. 555480), mouse anti-human CD19 (clone HIB19, BD Biosciences, cat. no. 562294), mouse anti-human CD3 (clone HIT3A, BD Biosciences, cat. no. 555337), and rat anti-mouse CD45 (clone 30-F11, BD Biosciences, cat. no. 553076]; mouse anti-human CD45 (clone HI30, BD Biosciences, cat. no. 555482), mouse anti-human CD45RA (clone HI100, BD Biosciences, cat. no. 555489), mouse anti-human CD25 (clone M-A251, BD Biosciences, cat. no. 562403), mouse anti-human CCR7 (clone G043H7, BioLegend (San Diego, CA, USA), cat. no. 353220), mouse anti-human CD45RO (clone UCHL1, Miltenyi Biotec, cat. no. 130-113-551, Cologne, Germany), mouse anti-human CD62L (clone DREGG56, BD Biosciences, cat. no. 559772), mouse anti-human CD8 (clone SK1, BD Biosciences, cat. no. 565192), FVS780 (BD Biosciences, cat. no. 565388), mouse anti-human CD3 (clone HIT3a, BD Biosciences, cat. no. 740073), and mouse anti-human CD4 (clone SK3, BD Biosciences, 562970)]; mouse anti-human CD45 (clone HI30, BD Biosciences, cat. no. 555482), mouse anti-human CD56 (clone B159, BD Biosciences, cat. no. 555516), mouse anti-human CD19 (clone HIB19, BD Biosciences, cat. no. 562294), mouse anti-human CD38 (clone HB7, BD Thermofisher Scientific, cat. no. 46-0388-42), mouse anti-human CD27 (clone M-T271, Miltenyi Biotec, 130-113-631, Cologne, Germany), mouse anti-human CD10 (clone HI10a, BioLegend, cat. no. 312210), mouse anti-human CD33 (clone P67.6, BD Biosciences, cat. no. 659113), rat anti-mouse mCD45 (clone 30-F11, BD Biosciences, cat. no. 557659), mouse anti-human CD3 (clone HIT3a, BD Biosciences, cat. no. 740073), and mouse anti-human CD16 (clone 3G8, BD Biosciences, cat. no. 563830)].

At various time points post-humanization surgery, blood was collected via retro-orbital bleeding and processed as previously described by Del Rio et al. (7) Then, 2 μL of FcR blocking reagent was added to each blood sample, which was then vortexed and incubated at room temperature for 2 min in the dark. Antibody master mix was subsequently added directly to each blood sample, which was vortexed and incubated at room temperature for 15 min in the dark. Then, 1 mL of BD Lysis Buffer (BD Biosciences, cat. no. 555899) was added to each sample, and the samples were incubated at 37 °C for 3 min. Samples were then centrifuged at 300 x g for 5 min. The supernatant was aspirated, leaving approximately 100 μL of each sample. Then, 1 mL of room-temperature FACS buffer was added to each sample, which was then vortexed and centrifuged at 300 x g for 5 min. A second wash step was then repeated as previously described. After the second wash, the supernatant was aspirated, leaving approximately 100 μL of each sample, which was then vortexed to break up the pellet. The samples were then analyzed using a Cytoflex Flow Cytometer (Beckman Coulter, Beckman Coulter). Doublets were removed using FSC-H vs FSC-A and SSC-H vs SSC-A gating.

Some cohorts of mice were assessed for human chimerism using the following method. At various time points post-humanization with CD34+ HSPCs, peripheral blood was collected via submandibular bleeding into K2EDTA-coated microtainers (Beckton Dickinson, San Jose, CA) and mixed to prevent clotting. Then, 75 µL of the blood sample was dispensed into a well of a 96-well deep well plate (Thermo Fisher Scientific). The blood samples were first labeled with Zombie Aqua fixable viability stain (BioLegend) for 20 min at room temperature. Cells were washed with 300 µL FACS buffer (1X PBS [VWR Scientific, Radnor, PA], 2% fetal bovine serum [Thermo Fisher Scientific], and 0.1% sodium azide [Sigma Aldrich]), and centrifuged at 310 x g for 5 min at 4 °C. Red blood cells were lysed by two 3.5-min incubations with 300 µL BD FACS lysing solution (Beckton Dickinson). After red blood cell lysis, samples were washed with 150 µL FACS buffer and spun at 310 x g for 5 min at 4 °C. The cells were resuspended in 150 µL FACS buffer containing the following fluorophore-conjugated antibodies: anti-mouse CD45 (clone: 30-F11, 103132); anti-human CD45 (clone: HI30, 304024); anti-human CD19 (clone: HIB19, 302206); anti-human CD3 (clone: UCHT1, 300439); anti-human CD4 (clone: SK3, 344642); anti-human CD8 (clone: SK1, 344744); anti-human CD33 (clone: P67.6, 366612); anti-human CD14 (clone: 63D3, 367144); anti-human CD16 (clone: 3G8, 302044); anti-human CD66b (clone: G10F5, 305116); and anti-human CD56 (clone: QA17A16, 392404) (BioLegend). Anti-HLA-A2 (clone BB7.2; BD Biosciences, Milpitas, CA, USA) was used for HLA disparity assays. Cells were incubated with antibodies at 4 °C for 40 min. Samples were washed with 150 µL FACS buffer, spun at 310 x g for 5 min, and resuspended in 150 µL BD Stabilizing fixative (Beckton Dickinson). After a 20-min incubation at 4 °C, cells were washed again in 150 µL FACS buffer. Finally, cells were resuspended in 400 µL FACS buffer and stored at 4 °C prior to analysis.

Prior to flow cytometry, 20 µL CountBright Absolute Counting Beads (Thermo Fisher Scientific) was added to each well for absolute cell count calculations. Calculations were performed according to the manufacturer’s instructions. Flow cytometry was performed using a Cytek Aurora (Cytek Biosciences, Fremont, CA) and analyzed using FlowJo Version 10 software (Beckton Dickinson). Human immune cell reconstitution was calculated as the percentage of hCD45+ cells among the total live single-cell population. Conversely, murine immune cells were calculated as mCD45+ cells within the same total live single-cell population. The human immune cell populations identified were: B cells (hCD45+, hCD19+); T cells (hCD45+, hCD3+, hCD56-); CD4+ T cells (hCD45+, hCD3+, hCD56+, hCD4+); CD8+ T cells (hCD45+, hCD3+, hCD56+, hCD4+); NK T cells (hCD45+, hCD3+, hCD56+); myeloid cells (hCD45+, hCD33+); monocytes (hCD45+, hCD33+, hCD66b-, hCD14+/hCD16+); granulocytes (hCD45+, hCD33+, hCD66b+); and NK cells (hCD45+, hCD19-, hCD3-, hCD33-, hCD56+/hCD16+).

Removal of sub-threshold engrafting mice is commonly reported in the HIS mouse literature. Although there is no universally accepted engraftment threshold, multiple groups report 10% hCD45^+^ as a minimum threshold for study inclusion (and >5% hCD3^+^ for inclusion in downstream studies) (35–37). Accordingly, we excluded mice from analysis that did not reach the 10% hCD45^+^ threshold, unless otherwise noted; the exclusion rate varied by cohort and was similar (approximately 10%) across the different HIS mouse models.

### Thymic graft analysis

Kidneys containing thymic grafts were harvested from euthanized humanized mice and stored in RPMI-1640 (Thermo Fisher Scientific, Waltham, MA) until processing. Euthanasia was performed by placing animals in an induction chamber that was then gradually filled with CO2 at a rate of 30%–70% volume per min until animals lost consciousness, with the flow rate then increased for >1 minute. Cardiac arrest was verified by palpation, followed by cervical dislocation as a secondary method to confirm euthanasia. The samples were rinsed in 1X PBS (VWR Scientific, Radnor, PA) and imaged on a Leica DMS 1000. Following imaging, thymic grafts were excised from the kidney and dissociated through a 70 µm cell strainer (Thermo Fisher Scientific, Waltham, MA) with the plunger of a 5 mL syringe (Beckton Dickinson). Cells were centrifuged at 300 x g for 5 min at 4 °C and then resuspended in 500 µL FACS Buffer. The entire volume was then moved to a well of a 96-well deep well plate (Thermo Fisher Scientific, Waltham, MA), and 150 µL BD FACS lysing solution was added (Beckton Dickinson) to lyse red blood cells. After a 3.5-min incubation, cells were spun at 310 x g for 5 min at 4 °C, and the red blood cell lysis was repeated. The samples were washed again in 150 µL FACS buffer and spun at 310 x g for 5 min at 4 °C. The cells were resuspended in 150 µL FACS buffer containing the following fluorophore-conjugated antibodies for 40 min at 4 °C: anti-mouse CD45 (clone: 30-F11, 103132); anti-human CD45 (clone: HI30, 304024); anti-human CD3 (clone: UCHT1, 300439); anti-human CD4 (clone: SK3, 344642); and anti-human CD8 (clone: SK1, 344744) (BioLegend). Samples were washed with 150 µL FACS buffer and centrifuged at 310 x g for 5 min. After removing the supernatant, the cells were resuspended in BD Stabilizing Fixative (Beckton Dickinson) and incubated for 20 min at 4 °C. After one additional wash with 150 µL FACS buffer, cells were centrifuged at 310 x g for 5 min and resuspended in 400 µL FACS Buffer. Samples were stored at 4 °C prior to analysis.

Prior to flow cytometry, 20 µL CountBright Absolute Counting Beads (Thermo Fisher Scientific, Waltham, MA) was added to each well for absolute cell count calculations. Calculations were performed according to the manufacturer’s instructions. Flow cytometry was performed using a Cytek Aurora (Cytek Biosciences, Fremont, CA) and analyzed using FlowJo Version 10 software (Beckton Dickinson). Human cells were identified as hCD45+ and calculated as a percentage of total single cells. The percentage of murine cells was calculated as the percentage of mCD45+ cells of total single cells. The human CD3+ and CD3neg cell populations were gated using CD4/CD8 as CD4+ single positive, CD8+ single positive, CD4+CD8+ double positive, and CD4negCD8neg- double negative to identify stages of T-cell thymic development.

### Single-cell RNA sequencing

T-cell gene expression was assessed using the 10X Genomics Chromium Single Cell 5′ Barcode Enabled Antigen Mapping (BEAM-T) kit following the manufacturer’s instructions (10X Genomics, cat. no. 1000539, Pleasanton, CA, USA). Raw sequencing data were processed using Cell Ranger (38) (10X Genomics, version 8.0, Pleasanton, CA, USA) for gene expression alignment to the human reference genome (GRCh38) and V(D)J alignment utilizing the GRCh38 alts ensemble-7.1.0 reference.

Downstream computational and statistical analysis was performed in R (version 4.2.3) utilizing the Seurat package (39) (version 5.0.1). To normalize the data and identify highly variable features, reads were regularized using a regularized negative binomial regression model (SCTransform (40)). During this step, the percentage of mitochondrial transcripts was regressed out to mitigate technical variance driven by cellular stress or apoptosis.

Dimensionality reduction was performed using Principal Component Analysis (PCA), using the first 50 principal components. Uniform Manifold Approximation and Projection (UMAP) was subsequently applied for non-linear dimensional reduction and two-dimensional visualization. To identify transcriptionally related cellular populations, graph-based clustering was performed using the Louvain algorithm across multiple resolutions, with a resolution of 0.8 used in the manuscript figures. Filtering thresholds for removal of transcripts were: >25% mitochondrial transcripts, >30,000 transcripts, <1000 transcripts, >50,000 unique features, and <250 unique features. The total cells recovered per sample, pre-filtering, were as follows: NeoThy Vax (6854), NeoThy Vehicle (16,329), Cord-Oly Vax (9688), and vaccinated human control (4858). No additional batch correction was performed beyond the variance-stabilizing transformation applied through SCTransform using a regularized negative binomial regression model. Finally, probabilistic cell-type annotation was conducted using Azimuth against the comprehensive peripheral blood mononuclear cell reference dataset (41).

Following annotation, differential gene expression analysis between experimental cohorts and cell types was performed using the FindMarkers function within the Seurat package. This function utilizes a Wilcoxon rank sum test to identify genes with statistically significant changes in expression. To control for multiple comparisons, p-values were adjusted using the Bonferroni correction. Genes were considered differentially expressed if they exhibited a Bonferroni-corrected p-value of less than 0.01. Additionally, targeted analysis of the TCR repertoire was conducted using the scRepertoire package (42) (version 2.0.0) in R. Filtered contig annotations generated by Cell Ranger were integrated with the existing Seurat single-cell gene expression objects to link clonotype data with individual cellular phenotypes. We utilized scRepertoire to perform clonal diversity analyses assessing repertoire breadth and oligoclonality across cohorts and to generate corresponding visualizations of TCR clonal expansion and distribution.

### Ex vivo T cell proliferation

Splenocytes were isolated from NeoThy mice at 24 weeks post-humanization. Spleens were gently dissociated through a 100 µm cell strainer (BD Biosciences, San Jose, CA) using the flat end of a sterile syringe plunger into Immunocult XF serum-free medium (Stem Cell Technologies, Vancouver, BC, Canada). The resulting suspension was subjected to density gradient separation using Lymphocyte Separation Medium (Corning, Manassas, VA). Red blood cells were removed by brief incubation in ACK lysis buffer (Thermo Fisher Scientific, Waltham, MA). Live cells were counted on a hemocytometer after staining with trypan blue. Following enumeration, cells were washed, resuspended in cold FACS buffer, and passed through fresh strainers as needed to eliminate remaining aggregates.

For plate stimulation, 48-well plates (Corning, Manassas, VA) were used. Splenocytes were labeled with VPD-450 dye (BD Biosciences) at 1 µL of a 1 mM stock diluted in dPBS-/- to obtain a 1uM working solution for a 1:1000 v/v dilution for 10–15 min at 37 °C. Cells were quenched with Immunocult XF medium (Stem Cell Technologies, Vancouver, BC, Canada) containing 20% FBS (Hyclone, Pittsburgh, PA) and washed twice more with dPBS-/-. CFSE-labeled cells were adjusted to 2 × 10^6^ cells/mL in Immunocult XF serum-free medium supplemented with 5% FBS, Penicillin–Streptomycin, and GlutaMAX (Thermo Fisher Scientific). A total of 500 µL of the cell suspension was added to each well, stimulated with Immunocult human T-cell activator (anti-CD3/anti-CD28) with 50IU/mL recombinant human IL2 (PeproTech, Rocky Hill, NJ), with cells alone without activator serving as unstimulated controls. Media were not replaced during the culture. Cell proliferation and morphology were monitored using an ECHO Revolve microscope, and proliferation was assessed by flow cytometry.

### Statistical analysis

Analysis of variance was used for comparing groups. Two-tailed Student’s t-tests (α=0.05 significance level) were used for comparisons of two samples with an assumption of equal variance. Where the assumption of equal variance was violated, we used a t-test assuming unequal variance. Ordinary one-way ANOVA analysis with Tukey’s multiple comparisons test was used when comparing multiple samples. Simple linear regression was performed to evaluate the relationship between thymic donor age and immune cell chimerism, with the coefficient of determination (R^2^) reported. P values less than 0.01 were considered significant. Analysis was performed with GraphPad Prism v10.2.2. (GraphPad Prism software, La Jolla, CA).

## Results

We have established an extensive cryobank of HLA-typed neonatal and pediatric thymus donors (**Supplementary Figure 1**), affording the opportunity to assess the impact of thymus donor age on T-cell development in NeoThy mice and to compare the NeoThy model to the BLT model (6, 7).

### Depletion of passenger thymocytes and de novo thymopoiesis

All BLT/NeoThy class HIS mice share a common design feature in that they are generated using immunodeficient mouse hosts that have been intravenously infused with cord blood CD34^+^ cells and then surgically implanted with human thymus fragments. Both of these interventions enable *de novo* human T-cell development (i.e., thymopoiesis) in the animals. Early BLT model iterations (4, 43, 44) used fresh (not frozen) tissues; however, a breakthrough 2012 publication by the Sykes group (45) demonstrated that human fetal CD34^+^ cells and thymus fragments could both be cryopreserved for later use in BLT mice. Additionally, they used an anti-CD2 antibody to remove fully-developed passenger thymocytes from the thymus graft, ensuring that only *de novo* T cells were produced in the model, rather than the mice also being engrafted with recent thymic emigrants that would presumably mediate graft-versus-host disease (GVHD) due to a lack of murine antigen exposure during their development. While not all investigators use antibody-based depletion in their BLT mice, our group adopted this approach for the development of the NeoThy model (6, 7), and we believe it is needed to ensure that passenger thymocytes are depleted from the transplanted thymus tissue and are not allowed to emigrate into circulation.

**Figure 1:**
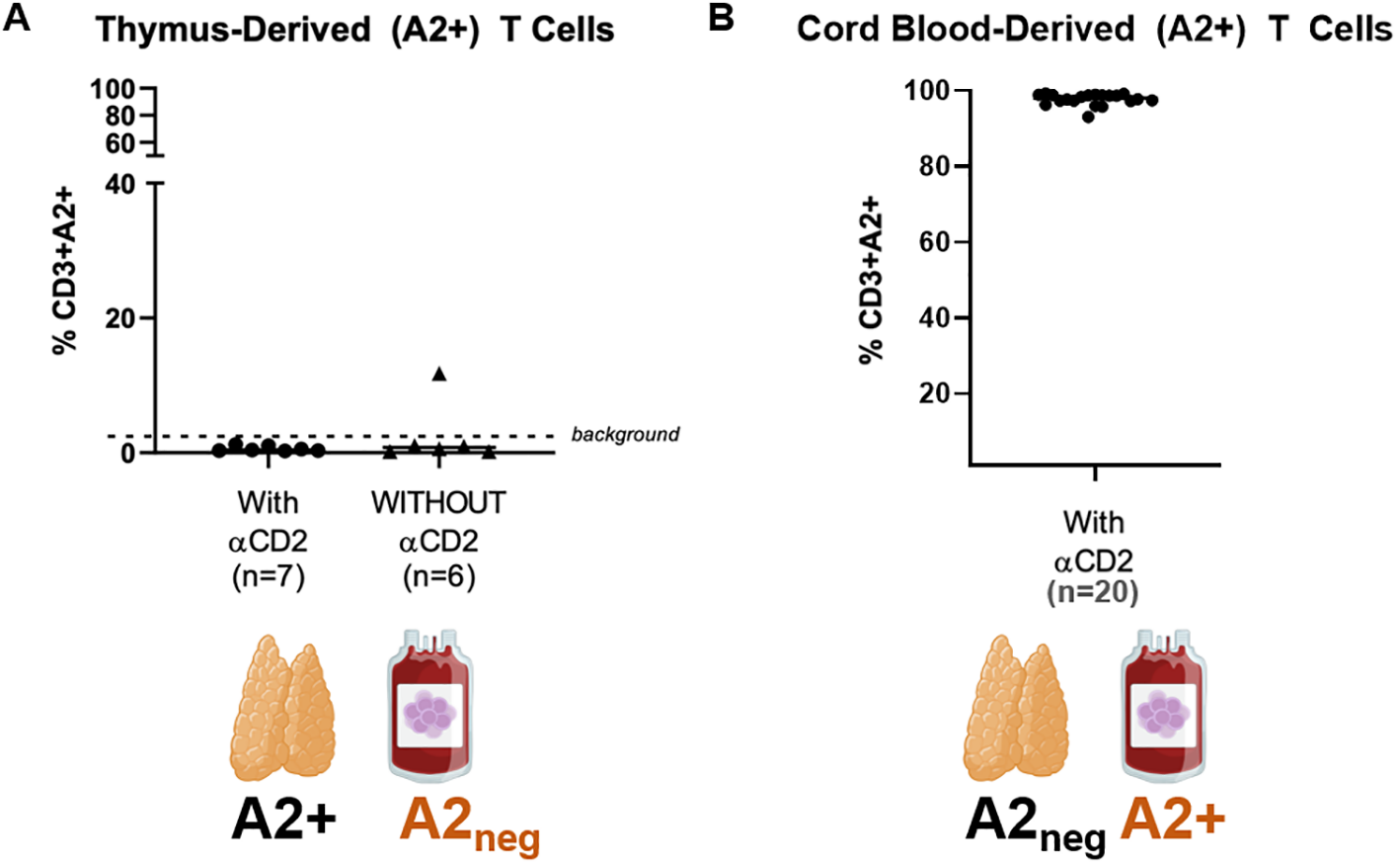
Assessment of *de novo* thymopoiesis vs passenger thymocyte repopulation via HLA disparity assay. **(A)** NeoThy mice were created using HLA-A2negative cord blood HSPCs and thymus fragments that were HLA-A2^+^ in order to track the origin of chimeric human T cells in circulation. One cohort of mice was created and administered antihuman CD2 antibody (n=7) and another cohort did not receive antibody at day 0 (n=6). Mice were bled at week 20w post-humanization and peripheral blood was analyzed with hCD3 and HLA-A2 co-staining by flow cytometry. Mice were >25% hCD45* and >20% hCD3^+^. T cells within the mice that were A2+ are an indication that the A2+ thymus was the source of the T cells. **(B)** A second cohort of NeoThy was created (n=20) using A2 thymus and A2^+^ cord blood; all were given aCD2 antibody treatment at day 0. Percentage (%) refers to the cell frequencies of the parent hCD45^+^ population. Analysis was performed via FlowJov10 software and plotted with GraphPad Prism10.2.2. Images created using Biorender.

Here, prior to conducting studies assessing the impact of thymus donor age on human chimerism in the NeoThy model, we first sought to verify *de novo* thymopoiesis and a lack of contribution of passenger thymocytes to the peripheral human T cell population in the mice. We utilized an HLA-specific antibody tracking method to identify the source (cord donor vs. thymus donor) of T cells in the mice. NeoThy mice were created using HLA-A2^+^ thymus donor tissue along with allogeneic HLA-A2^neg^ cord blood CD34^+^ cells (**Figure 1A**), as HIS mice can be made with allogeneic thymus and cord donors with varying degrees of HLA matching, including total major histocompatibility complex (MHC) mismatch (**Supplementary Figure 2**). Cohorts of mice were generated with and without administration of anti-CD2 T cell-depleting antibody (100 ug per dose in two doses on Day 0 and Day 7 post-surgery) in order to validate antibody-based depletion and verify T cell origin. Without the anti-CD2 antibody, one of six mice showed HLA-A2^+^ (thymus donor passenger thymocyte-derived) human CD3^+^ T cells in the periphery, whereas none of the antibody-injected animals showed passenger thymocyte-derived T cells. We then changed donors to reverse the labeling paradigm, using an HLA-A2^neg^ neonatal thymus donor along with HLA-A2^+^ cord blood CD34^+^ cells to create NeoThy mice. All the HIS mice showed HLA-A2^+^ human CD3^+^ T cells and no appreciable number of HLA-A2^neg^ cells above background noise when given antibody treatment (n = 20 mice, **Figure 1B**). These results demonstrate that the depletion antibody protocol is effective at ensuring only *de novo* T cells are present in the NeoThy mice.

**Figure 2:**
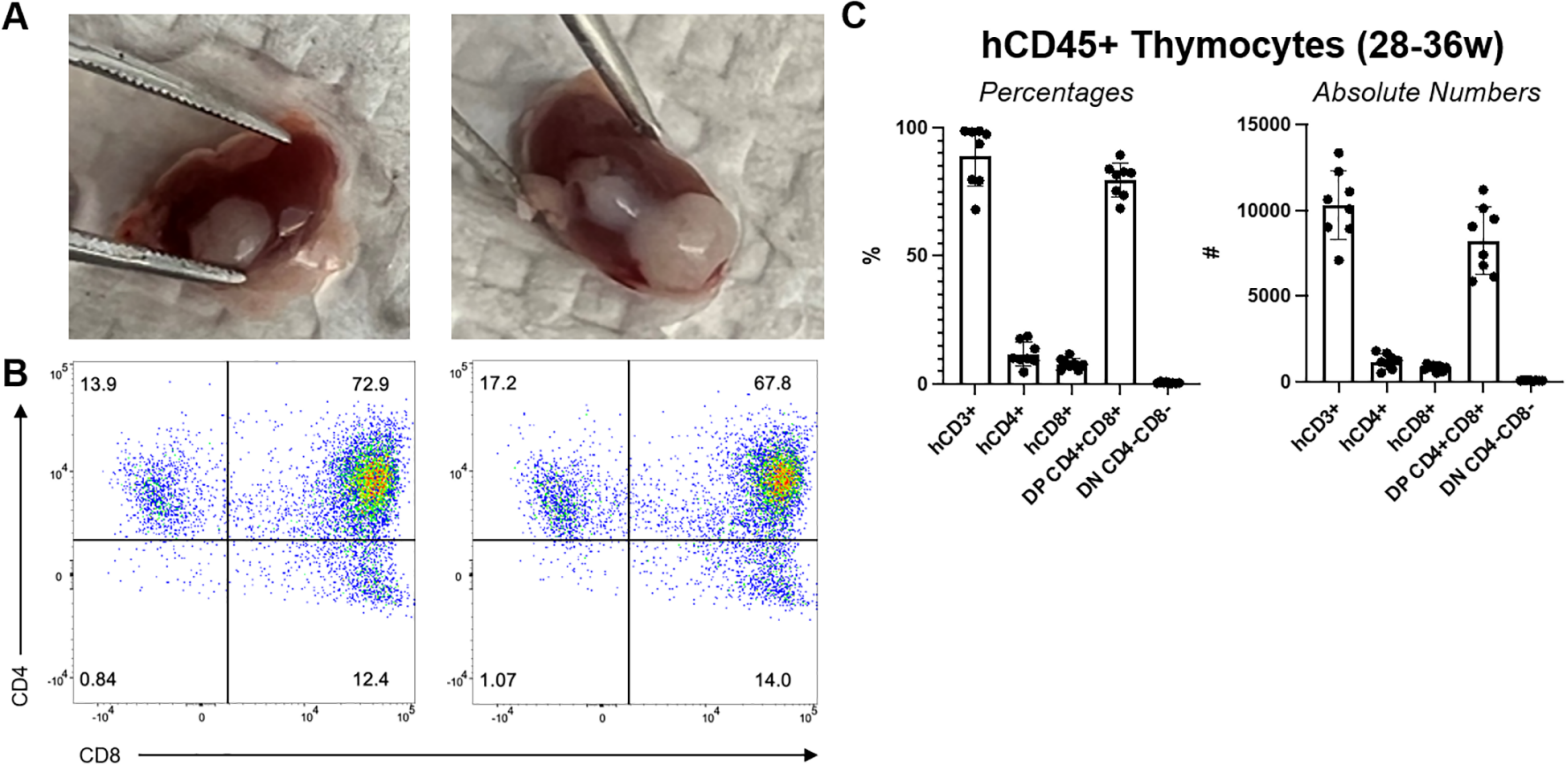
Thymopoiesis in the NeoThy mouse human thymic graft. **(A)** Representative images of NeoThy human thymic grafts in the kidney are shown. **(B)** Developing thymocytes were isolated from grafts via dissociation and straining of tissue, then stained for T cell markers and analyzed by flow cytometry. Of the CD3^+^ cells, Double Positive (CD4^+^CD8^+^), Single Positive CD4^+^, and Single Positive CD8^+^ cells are shown in representative flow plots. **(C)** Data from multiple experiments and donors (n=4; 7d, 14d, 232d, and 300d old donors) in the 28-36 weeks post-humanization timeframe were compiled (total of n=17 thymic grafts assessed). Percentage (%) and number (#) refer to the cell frequencies of the parent hCD45+ population. Data are plotted with GraphPad Prism 10.2.2, with flow cytometry data analyzed via FlowJo v10 software.

Next, neonatal thymic explants were recovered from NeoThy mice at 28–36 weeks post-humanization from donors of multiple ages (n = 4; 7-day-old, 14-day-old, 232-day-old, and 300-day-old donors). Grafts varied in size (**Figure 2A**); grossly apparent grafts were excised, mashed through sterile cell strainers, and stained for human (h) CD45, hCD3, hCD4, and hCD8 markers to evaluate the presence of developing double-positive (DP) and single-positive (SP) T cells within the graft. Characteristic DP, CD4^+^ SP, and CD8^+^ SP thymocytes (**Figure 2B**) were apparent in the thymic grafts across multiple donors (**Figure 2C**). These results demonstrate *de novo* T-cell development occurring within the transplanted thymus grafts of the NeoThy model.

### Immune cell engraftment in fetal vs. non-fetal HIS mice

Having established evidence of *de novo* T cell development originating from the input cord blood CD34^+^ HSPCs educated by the thymic epithelial cells of the graft, rather than the T cells being present due to emigration of passenger thymocytes present at the time of humanization surgery, we next investigated the impact of thymic donor age on human immune cell reconstitution.

**Figure 3:**
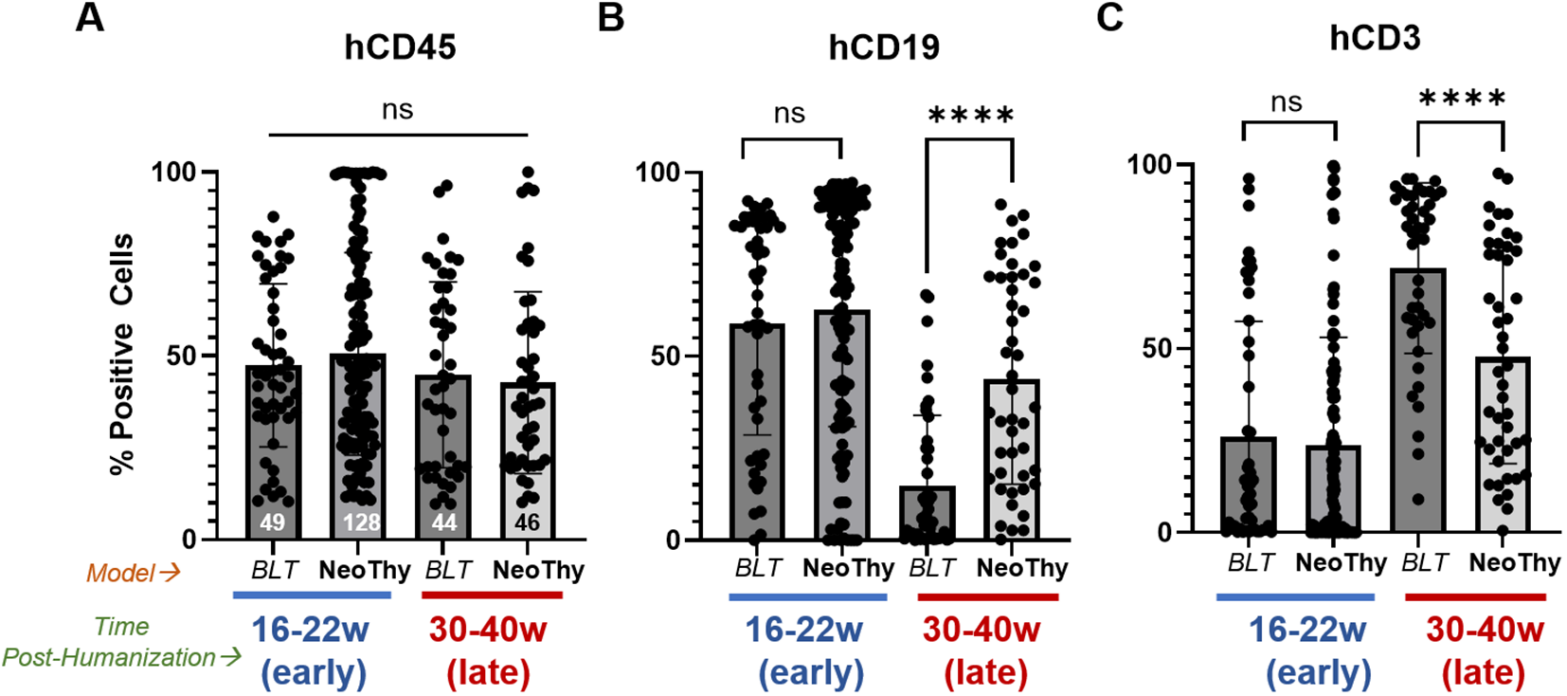
Human immune chimerism in BLT vs NeoThy mice with young thymic donor tissue. **(A)**. BLT mice were made using 16-18 weeks gestational age fetal tissue and compared to NeoThy mice made from younger neonatal thymus donors (0-<4 months old) and cord blood HSPCs for **(A)** total hCD45+ chimerism, **(B)** hCD 19+ B cells (of the hCD45+) and hCD3^+^ T cell (of the hCD45+) subsets at early (16-22 weeks post-humanization) and late (30-40 weeks post-humanization) timepoints. Mouse Nvalues for each condition are shown at the base of the bars in **(A)**. Percentage (%) Positive Cells refers to the cell frequencies of the cells in the total (mCD45+ hCD45) or parent hCD45 population. Data are from pooled donors (n=8 for BLT, n=11for NeoThy). Data are plotted and statistics conducted with GraphPad Prism 10.2.2. Unpaired two-tailed ttests were conducted using Welch's correction. ****p<0.0001. Coefficients of variation (CVS) for hCD3 were 119.9% (BLT 16-22w), 123.1% (NeoThy 16-22w), 32.25% (BLT 30-40w), and 60.71% (NeoThy 30- 40w). Flow cytometry data analyzed via FlowJo v10 software. ns, not significant.

First, we conducted comparative studies of fetal tissue-derived BLT mice of consistent 16–18 week gestational age versus NeoThy mice generated from “young” neonatal thymus donors (0 to <4 months old) and cord blood. BLT mice (n = 8) using fetal tissue at a set gestational age and NeoThy mice (n = 11) generated from young donor thymus tissue were assessed for human chimerism at multiple time points. Overall, hCD45^+^ leukocyte chimerism was similar between the groups, with no statistically significant differences at early or late time points post-humanization across all fetal and non-fetal thymus donors (**Figure 3A**). Of the hCD45^+^ cells, hCD19 B cell (**Figure 3B**) and hCD3^+^ T cell (**Figure 3C**) frequencies in the mice were similar at the early time point (for B cells: 58.8 +/- 30.2 for BLT mice and 62.7 +/- 31.8 for NeoThy mice; and for T cells: 26.1 +/- 31.3 for BLT mice and 23.8 +/- 29.3 for NeoThy mice). However, at the later time point, the BLT mice showed a higher mean T-cell frequency (71.9 +/- 23.2) than NeoThy mice (47.7 +/- 29.0), whereas B cells followed an inverse relationship (14.9 +/- 19.1 in BLT mice and 43.8 +/- 28.5 in NeoThy mice). As this class of model is designed to be T-cell centric (5, 43) and B cells and other compartments are noted to be less physiologically relevant in HIS models (46), we focused on T cells for downstream analysis. It should be noted that the T-cell frequencies observed at the later time point in the BLT model reflect supraphysiologic levels (i.e., higher than are typically seen in healthy children and adults) (47, 48). The NeoThy mice replicated our previously published report of a more gradual emergence of hCD3^+^ T cells and levels that are more physiologically representative of healthy children and adults, which is in line with the original goals of developing these T-cell-centric models (6). Despite elevated hCD3^+^ levels at the later time point in BLT mice, >90% of both the BLT mice and young donor NeoThy mice engrafted >10% hCD3^+^ cells by the late time point regardless of the age disparity (age difference of approximately 5–7 months from fetal to newborn) between the fetal and non-fetal donors, meeting a threshold used in multiple HIS model publications for downstream use (35–37).

### Impact of non-fetal thymic donor age on T cell chimerism

**Figure 4:**
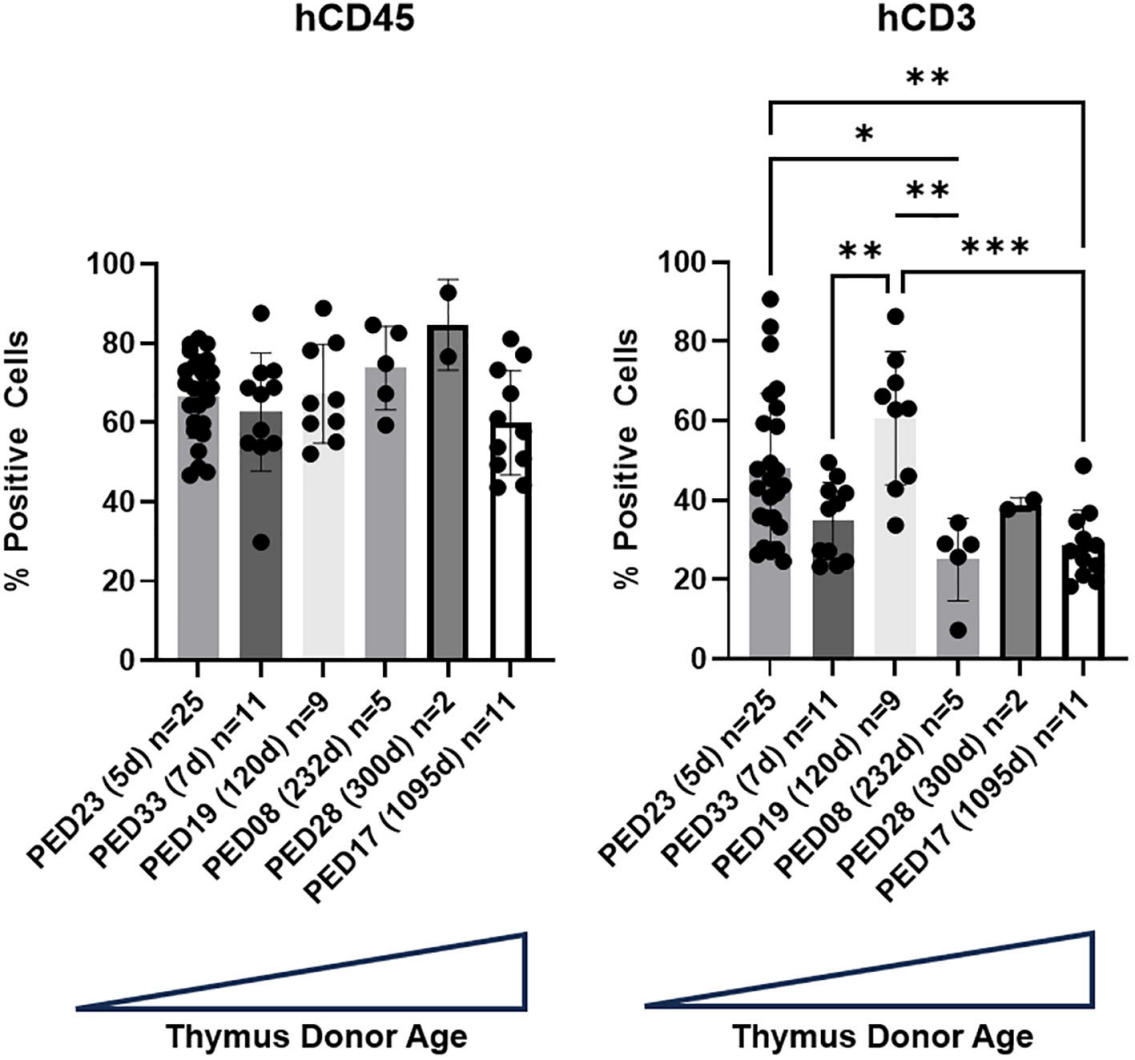
NeoThy mouse chimerism from cohorts created using thymic donors of varying age. NeoThy mice were made using cryopreserved and HLA-typed thymus fragments from young donors (0-<4 months old), mid-age donors (4m - <7m), and older donors (7m-12 years) plus cord blood in myeloablated immune deficient NOG-IL6 mice. **(A)** Mice were assessed by flow cytometry for total hCD45^+^ (left) chimerism and hCD3^+^ T cells (right). Percentage (%) Positive Cells refers to the cell frequencies of the cells in the total (mCD45^+^ hCD45) or parent hCD45 population. Mice that did not meet>25% hCD45 threshold by week 16 were excluded from the study. Data are from pooled donors at the approximately 22w timepoint is shown. Data are plotted and statistically analyzed with GraphPad Prism 10.2.2. One-way ANOVA with Tukey's multiple comparisons test and simple linear regression analysis was conducted, all differences were not significant in hCD45 (all >0.1) and were not significant for hCD3 unless noted. For hCD45, R2 = 0.026; for hCD3, R2 = 0.127. * is p<0.1, ** is p<0.01, *** is p<0.001. Coefficients of variation (CVS) for hCD3, in order of donors left to right, were: 38.86%, 28.06%, 27.66%, 41.59%, 4.36%, and 31.26%. Flow cytometry data analyzed via FlowJo v10 software.

We next evaluated NeoThy mice generated from donors of different age groups, including “young” neonatal (0 to <4 months; n = 36),”older” pediatric (>7 months to 12 years, n = 18) donors, and “intermediate” (>4 to <7 months, n = 9) thymic donors. As mentioned above, one previous study reported some evidence of diminished T-cell chimerism with increasing thymic donor age (25), so we established cohorts of mice from thymic donors within each of these above age ranges and then assessed for overall hCD45^+^ and hCD3^+^ chimerism (**Figure 4**). No statistically significant relationship between donor age and hCD45 chimerism was observed (R^2^ = 0.026). While there were statistically significant frequencies of T cells between some age cohorts via ANOVA (**Figure 4**, right), the values had only a modest inverse trend (R2 = 0.127) and did not reach statistical significance, i.e., it was substantially outweighed by inter-donor variability. Frequencies of hCD3^+^hCD4^+^ and hCD3^+^hCD8^+^ T cells were also variable across donors, with no statistically significant differences directly correlated with donor age (**Supplementary Figure 3A**). There were also no differences in naïve T cells (**Supplementary Figure 3B**) that rose above donor-to-donor variability.

### Assessment of T cell function

As mentioned in the Introduction, HIS mouse models have traditionally been used for a wide range of research applications, including transplantation and regenerative medicine (8), infectious diseases (e.g., EBV (4) and HIV (9)), toxicology (10), and immuno-oncology (11, 12). Each of these applications utilizes subject area-specific functional assays, and it is therefore not possible to interrogate the role of thymus donor age in every possible experimental context. Instead, we sought to evaluate *in vivo* cytokine secretion and *ex vivo* T-cell proliferation in the NeoThy model to provide important generalizable data related to T-cell function pertinent to multiple research contexts.

**Figure 5:**
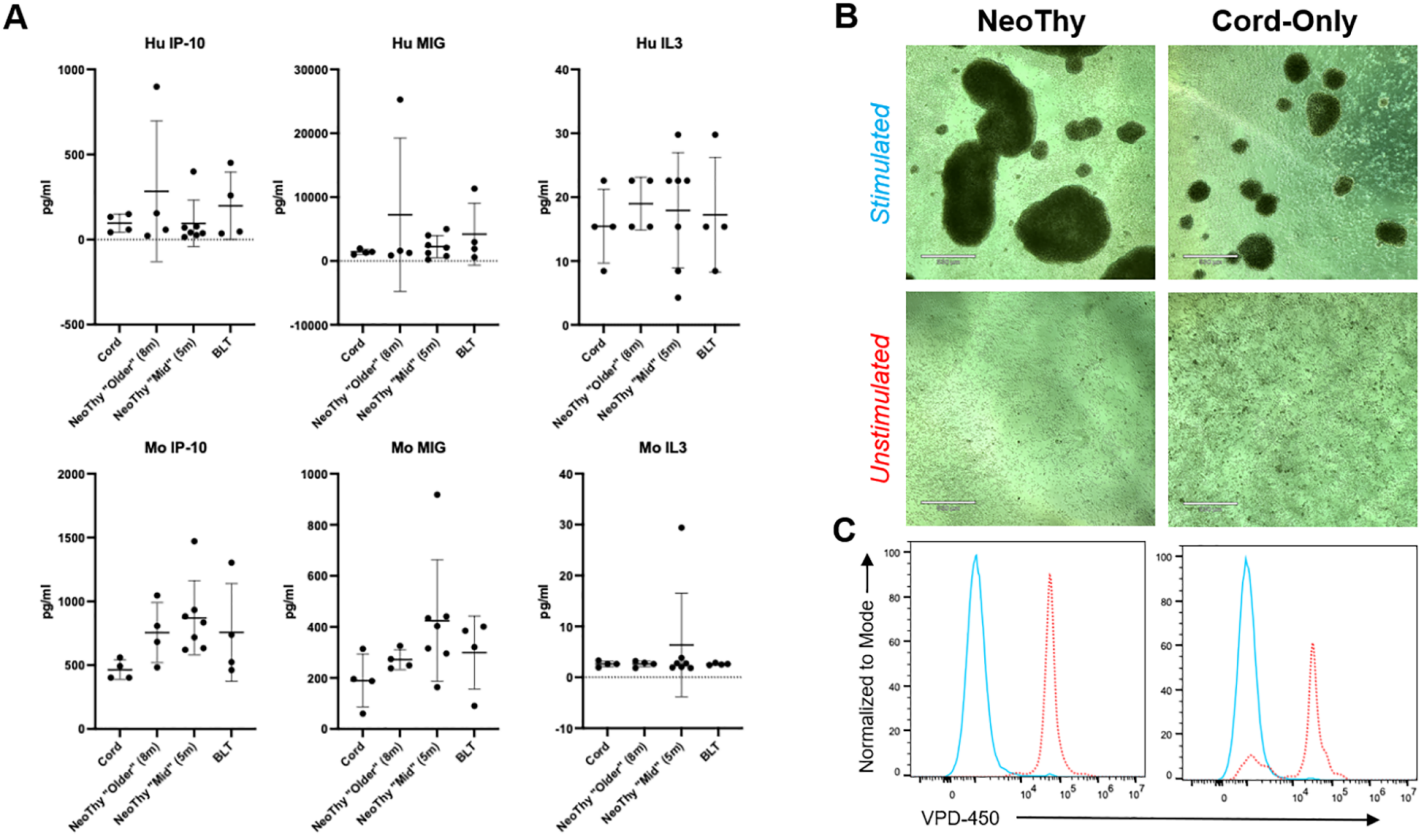
Assessment of T cell functional proliferation and cytokine secretion. NeoThy mice were made in the NBSGW strain from young (0-<4 months old, n=4) and older (7m-12 years, n=7) thymus donors and compared to cord blood only humanized mice (made from a common cord blood donor with the older mice, n=4) and BLT mice (n=4). **(A)** Mouse serum was collected at 17-26 weeks post-humanization and assessed via the MilliporeSigma Humanized Mouse Cytokine Assay for 41 human-and mouse-specific cytokines. Representative plots of human (top) and mouse cytokines (bottom) are shown. **(B)** Mice were euthanized at 24 weeks post-humanization, splenocytes were harvested, stained with VPD-450 proliferation dye, stimulated with anti-CD3/anti-CD28 beads, and cultured with rhlL2 in serum- free medium for 6 days. Proliferative T cell blasts were observed via phase contrast microscopy and **(C)** assessed by flow cytometry for T cell proliferation; unstimulated cells in red, stimulated cells in blue. Data are plotted and statistics conducted with GraphPad Prism 10.2.2. One-way ANOVA with Tukey's multiple comparisons test and unpaired two-tailed t tests with Welch's correction were conducted, with no differences rising to the level of statistical significance in any condition (all p values were >0.1). Flow cytometry data analyzed via FlowJov10 software. Images were acquired on ECHO Revolve | R4 microscope.

Serum samples were collected from NeoThy mice generated using intermediate (4 to <7 months, n = 4) and older (>7 months, n = 7) donor ages. The mice secreted human cytokines, including IP-10, MIG, and IL3 (**Figure 5A**), into the mouse circulation, similar to the BLT model and cord blood-only model controls, as assessed using a Luminex Humanized Mouse Cytokine Assay capable of species-specific detection of human versus mouse-derived factors, at a terminal 24-week time point. No significant differences in human or mouse cytokines were shown in any class of humanized mice. Notably, there were outlier mice expressing high levels of human (**Supplementary Figure 4**) and mouse cytokines (**Supplementary Figure 5**) without any statistically significant or otherwise discernible pattern associated with model type or thymic donor age.

As a test of T cell function in NeoThy animals, splenocytes were harvested by first passing excised spleens through sterile 100 µm mesh cell strainers, followed by lysis of red blood cells and washing. Individualized cells were then stimulated with anti-CD3/anti-CD28 beads and cultured with rhIL2 in serum-free medium for 6 days. Blasting human T cells were similarly observed in all conditions (**Figure 5B**) and proliferated in response to TCR stimulation (**Figure 5C**).

To assess antigen-specific T cell functional responses, we considered leveraging vaccination studies using the keyhole limpet hemocyanin (KLH) antigen. KLH vaccination is a classic vaccination strategy used to demonstrate that the immune system is capable of mounting a T-cell-dependent response to a strong antigen. However, because KLH is a foreign protein not encountered by human patients, we sought a more clinically relevant vaccination approach. We therefore focused our vaccination efforts on using an mRNA vaccine targeting the SARS-CoV-2 spike protein. NeoThy mice generated using an older thymic donor tissue, i.e., 10-month-old donor, and cord blood-only HIS mice generated using the same cord blood donor as used with the NeoThy animals, were used to assess the impact of inclusion of the thymus tissue graft. Vehicle-alone injections served as vaccination negative controls. In contrast to KLH, which is a large protein (~390kDa) (49) that, following processing, can generate dozens to hundreds of distinct peptides, resulting in polyclonal immune responses, mRNA vaccines encode a single defined protein and a limited subset of peptides following processing. Furthermore, lipid nanoparticle-encapsulated mRNA vaccines have a self-adjuvanting effect due to both the mRNA and the ionizable lipid components activating innate immune pathways (50–52).

**Figure 6:**
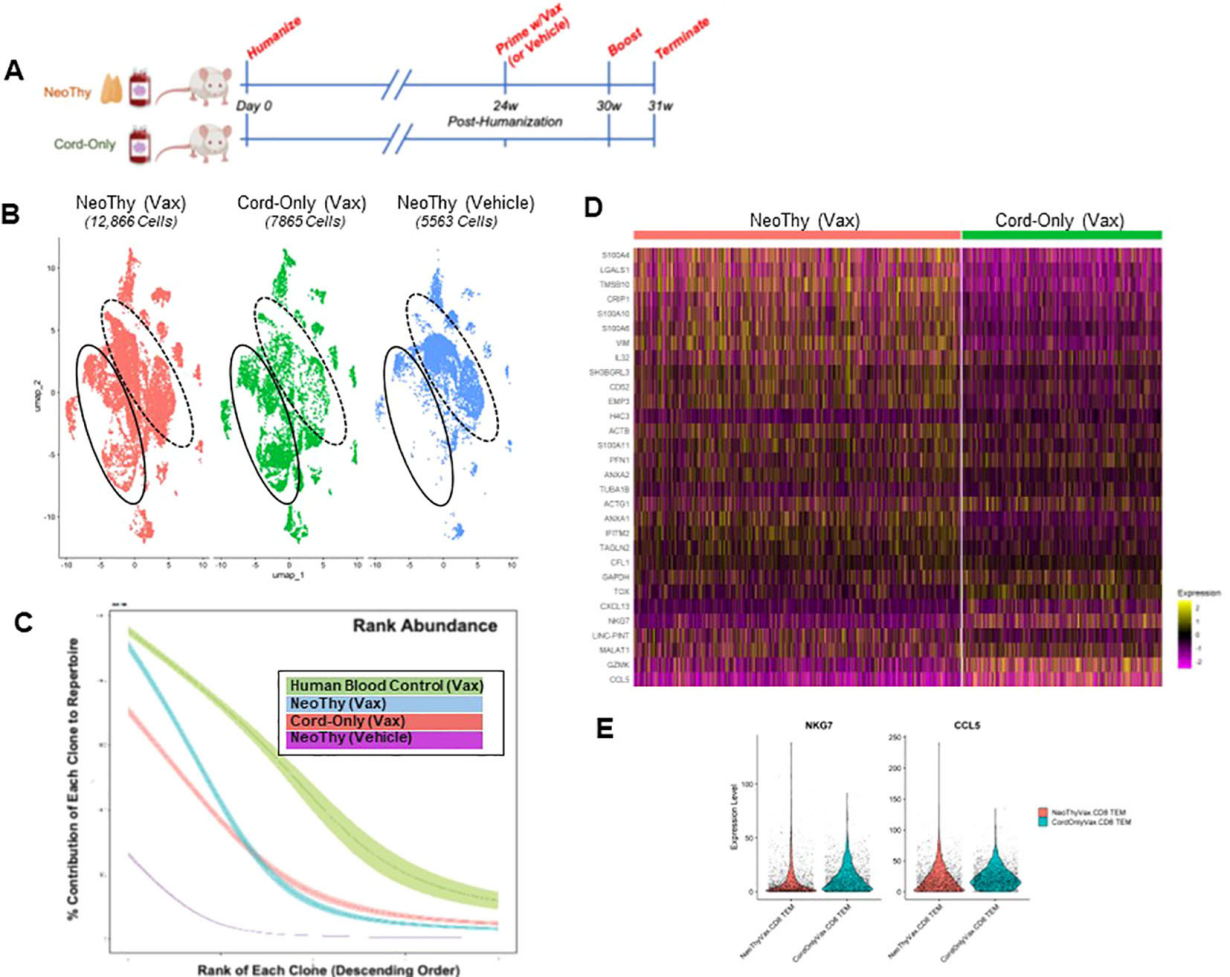
Vaccination of NeoThy mice with SARS-COV-2 spike mRNA. NeoThy mice were made in myeloablated immune deficient NOG-IL6 mice using cryopreserved and HLA-typed thymus fragments from a mid-age donor (5m). Thymus and cord blood were HLA matched at HLA-A*02, HLA-B7, HLA-C*07 and HLA-DRB1*15. Cord blood-only HIS mice were made using the same common cord blood donor as the NeoThy animals. **(A)** After verifying human T cell chimerism, n=5 HIS mice per condition were vaccinated (or received vehicle alone) at 24 weeks with a prime and boost 6 weeks later of SARS-CoV-2 spike protein encoding mRNA + lipid nanoparticle vaccine. At the terminal 31w timepoint, mice were sacrificed and splenocytes harvested for single cell RNA sequencing, one mouse per group with cell numbers denoted. **(B)** UMAPS show clusters of cells from vaccinated NeoThy and cord blood-only mice, compared to vehicle-alone (non- vaccinated) NeoThy mice. Solid circle denotes clusters associated with receipt of vaccine, dashed oval denotes clusters impacted by +/- of thymus transplant. **(C)** TCR repertoire clonality and repertoire diversity was assessed in the three conditions versus a healthy, vaccinated adult donor. **(D)** A heat map is shown of the top 30 most significant differentially expressed genes when comparing the two vaccinated models, each vertical line represents an individual cell. **(E)** Violin plots for genes of interest NKG7 and CCL5 expression are shown. Graphic created using Biorender.

We conducted a two-dose vaccination study (prime plus a boost 6 weeks later), assessing mice one week after the booster dose, based on previously published studies using the Moderna mRNA-1273 vaccine in immunocompetent BALB/c mice (**Figure 6A**) (53). In our study, mice were assessed for human chimerism at multiple time points to confirm >25% hCD45+ and >10% hCD3+ cell frequencies. At the terminal time point following vaccination, splenocytes were harvested and processed for single-cell RNA sequencing. Mice showed distinct UMAP clustering depending on whether they were vaccinated and whether they had received a thymic transplant (i.e., NeoThy mice vs cord blood-only mice) (**Figure 6B**). Despite having the same HSPC donor, vaccination in the cord blood-only (no thymic transplant) HIS context resulted in T cells with less TCR clonal diversity (**Figure 6C**). Hill diversity analysis confirmed broader and less oligoclonal repertoires, suggesting improved adaptive immune modeling in the presence of human thymic education. Vaccinated cord blood-only mice demonstrated upregulation of genes associated with cytotoxicity and dysfunctional or incompletely matured T cell states, including TOX, GZMK, CD74, and PECAM1. NeoThy mouse vaccination resulted in upregulation of antiviral and regulatory genes, including ZFP36, NFKBIA, ANXA1, and IFITM2, consistent with coordinated and moderated immune activation (**Figure 6D**). Interestingly, IFITM2 is an important antiviral membrane protein gene (54) associated with inhibited viral entry (55), enhancement of T cell immunity (56), and correlated with increased type 1 interferons following vaccination (57).

**Figure 7:**
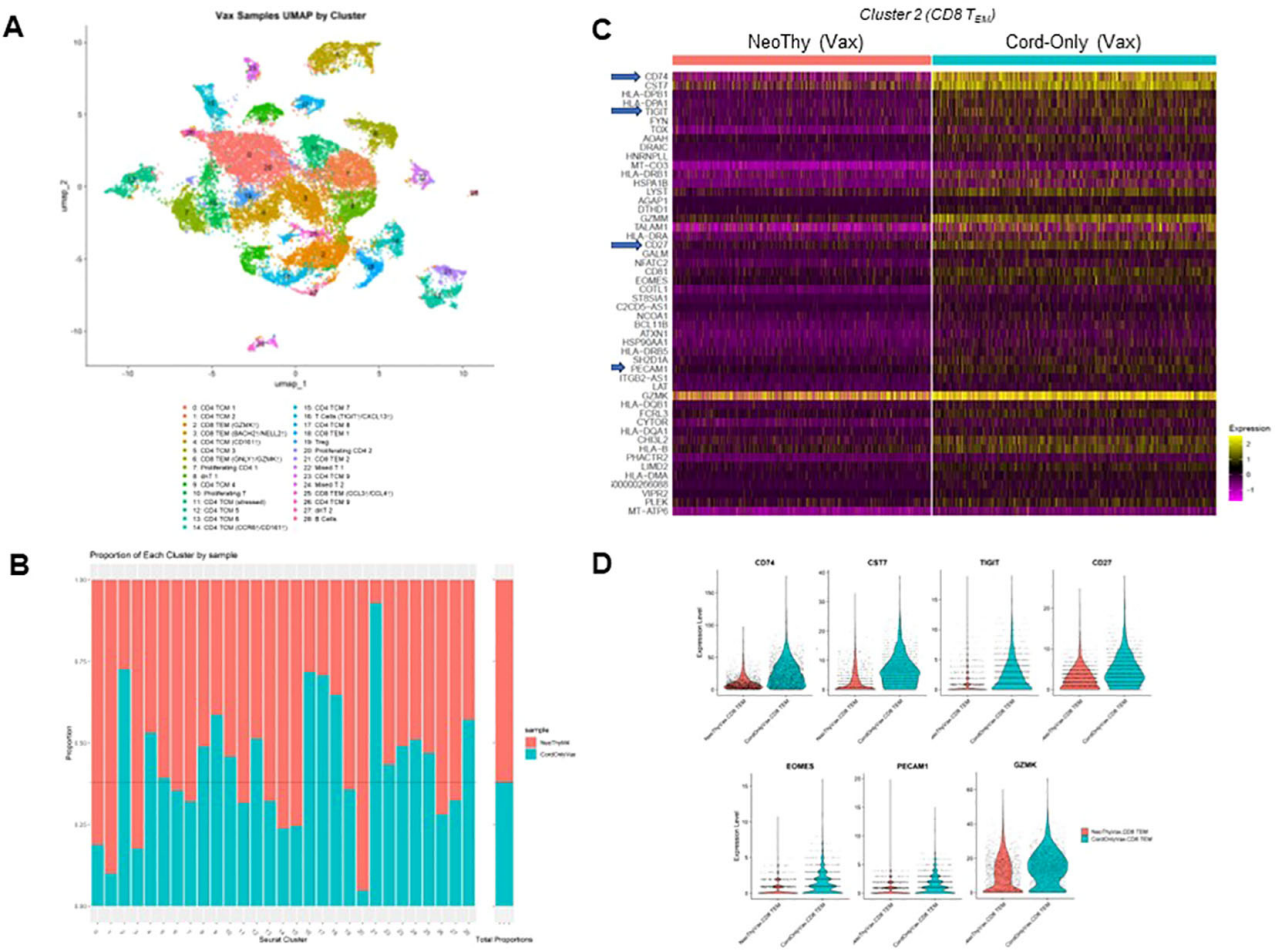
Assessment of T cell subsets following vaccination of NeoThy mice with SARS-CoV-2 spike mRNA. NeoThy mice were made in myeloablated immune deficient NOG-IL6 mice using cryopreserved and HLA-typed thymus fragments from a mid-age donor (5m). Thymus and cord blood were HLA matched at HLA-A*02, HLA- B7, HLA-C*07 and HLA-DRB1*15. Cord blood-only HIS mice were made using the same common cord blood donor as the NeoThy animals. Mice were vaccinated, see **Figure 6A** schematic (n=5 per condition) **(A)** UMAPS show computationally defined population clusters of cells from vaccinated NeoThy and cord blood-only mice; data shown are from one of each type of vaccinated mouse (12,866 cells for NeoThy, 7865 cells for cord blood-only). **(B)** Proportions of cells in each cluster in the two model types are show. Dashed line indicates theoretical proportion that would exist based on the assumption of equal distribution, in light of the total number of cells in the respective groups. Most common clusters are at left, going to the least common rightward. **(C)** A heat map is shown of the top 50 most significant upregulated genes in the CD8 effector memory T cell cluster from A (cluster 2) when comparing the two vaccinated models. Genes of interested are noted with arrows and reflected in **(D)** violin plots.

Upon reclustering of the vaccinated NeoThy mice and vaccinated cord blood-only mouse samples (**Figure 7A**), we identified specific clusters that were over- or under-represented in the two sample types (**Figure 7B**). Cluster 2 cells, which were largely comprised of CD8 effector memory T cells (T_EM_), showed a generally higher expression level of multiple genes in the cord blood-only condition, indicating aberrant gene expression. In particular, CD74 upregulation indicates altered antigen processing and presentation. PECAM1 is a marker of recent thymic emigrants in T cells (58), and the higher expression of that gene, together with CST7 and GZMM, indicates a less mature cell state (**Figures 7C, D**). Collectively, these findings indicate that the NeoThy animals receiving thymus transplantation, which confers human MHC-specific cues to the chimeric T cells during development, harbor a T cell repertoire that more closely resembles that of human patients in terms of their T-cell transcriptional profile response to vaccination.

We did not assess for spike protein-specific antibody due to the literature reporting low levels of antibody class switching in the HIS mice (59–61). We evaluated general antibody isotypes following the terminal time point. Although some mice exhibited apparent class-switched IgG antibodies (**Supplementary Figure 6**), these animals tended to be outliers. Overall, we observed sub-physiologic IgG levels, in line with previous reports in both BLT mice and cord blood-humanized mice (62). This effect could be attributable to the fact that one donor set was used for these experiments (i.e., there may be donor-to-donor differences). Additionally, the optimal time point for antibody assessment needs to be empirically determined because, similar to adaptive immune cell frequencies, antibody levels may change according to both time post-humanization and/or time post-vaccination.

## Discussion

In this study, we systematically examined the impact of thymic donor age and platform design on human immune reconstitution in thymus-based HIS humanized mouse models. Across multiple cohorts, we found no consistent differences in overall human leukocyte engraftment, T cell frequencies, *in vivo* cytokine expression, or broad *ex vivo* T cell proliferative function attributable to thymic donor ages spanning neonatal through pediatric sources. The comparative analysis of fetal-derived BLT mice and non-fetal NeoThy mice revealed largely similar immune reconstitution, with the notable exception of accelerated and often supraphysiologic peripheral T-cell expansion in the BLT model. Together, these data indicate that within the age ranges tested, postnatal thymic donor age is not a primary determinant of quantitative T cell chimerism. However, the presence of thymus clearly influences the quality of the chimeric human T cells (e.g., a more mature gene expression profile) relative to cord blood-only controls.

A critical foundation of this work was the definitive establishment of *de novo* thymopoiesis in the NeoThy model. Using HLA-mismatched tracking combined with anti-CD2-mediated depletion, we confirm and extend our prior reports (6, 7) demonstrating that passenger thymocytes can be effectively eliminated and do not contribute meaningfully to peripheral T cell pools when antibody treatment is employed. In parallel, recovery of thymic grafts containing canonical DP and SP thymocyte populations established that T cell development occurs within the human thymic graft itself. While we cannot completely exclude minor contributions from the murine thymus (an issue shared by most HIS models in the absence of thymectomy, including those used in this study (24, 63)), these data strongly support graft-centered human thymopoiesis as the principal source of T cells in NeoThy mice. Importantly, these findings underscore the need for harmonization of the NeoThy model and BLT model humanization methodologies, including cryopreservation strategies and passenger thymocyte depletion, when comparing studies across laboratories. Variability in such parameters may contribute to discrepancies reported in the literature (25), including differences between studies employing antibody-mediated depletion (45) and the majority that do not (13, 15, 16, 64, 65).

Our comparison of fetal BLT mice and neonatal NeoThy mice revealed broadly similar levels of hCD45 and hCD3 chimerism, but with supraphysiologic peripheral T cell expansion in the BLT model (which equates to higher frequencies of B cells in the NeoThy). In healthy human children and adults, circulating T cells typically comprise approximately 48%–56% of lymphocytes (47, 48); sustained elevations beyond physiologic norms may reflect altered homeostatic cues (66), thymic output dynamics (67), or peripheral expansion biases (68, 69). Whether the elevated T cell frequencies observed in BLT mouse cohorts represent enhanced effector function (4), dysregulated expansion (16), a fetal-biased repertoire (17), and/or influence functional assays (16, 70) remains unclear and warrants additional mechanistic investigation. Our findings suggest that quantitative T cell abundance assays alone are insufficient as a definitive performance metric and reinforce the importance of integrating functional and transcriptomic analyses when evaluating the fidelity of HIS models to adult human immunity.

A notable feature across cohorts was substantial donor-to-donor variability (i.e., variation in T cell frequency in a given donor that was not obviously associated with donor age) and the presence of outlier animals within cohorts. Rather than viewing these differences as a limitation, we interpret them as a reflection of authentic human biological diversity. Donor variability may offer an opportunity to model patient-specific immune features, particularly in contexts such as transplantation, regenerative medicine (including studies of induced pluripotent stem cell [iPSC]-based therapies (8, 22, 71, 72)), vaccine responses, and immune dysregulation. At the same time, for many applications, experimental robustness and reproducibility across donors remain essential. Future studies should therefore aim to distinguish which immune parameters represent stable platform attributes and which reflect donor-specific signatures. Large cryobanks of HLA-typed neonatal thymic tissue, such as those utilized here, uniquely enable controlled repeat experimentation, HLA matching strategies, genetic manipulation (including iPSC derivation and genetic editing/correction) (22), and longitudinal optimization studies while preserving donor traceability.

Functional assessment proved essential for distinguishing platform attributes that were not apparent by flow cytometry phenotyping alone. While overall T cell frequencies were roughly similar across age groups and did not scale proportionally with donor age, vaccination studies revealed novel qualitative differences in T cell transcriptomic states and TCR diversity metrics associated with thymus tissue in the NeoThy model. In our SARS-CoV-2 mRNA vaccine study, the NeoThy model demonstrated broader and less oligoclonal repertoires, along with antiviral and regulated activation signatures (e.g., IFITM2, ZFP36, and NFKBIA), whereas cord blood-only humanization (from the same donor but lacking thymic transplantation) resulted in transcriptional features associated with cytotoxic skewing and dysfunctional or incompletely matured states (e.g., TOX and GZMK). Importantly, these findings highlight a critical aspect of humanized mouse studies, namely, that in-depth transcriptomics studies can reveal differences among models that might not be apparent through general flow cytometry phenotyping.

Antibody levels alone did not distinguish platforms, consistent with known limitations in class switching in HIS models. However, similar to comparisons that rely on frequency alone as a measure of T cell capabilities, antibody quantity alone may not be the best metric for evaluating HIS models. Instead, antibody specificity, avidity, and functional capabilities—which were beyond the scope of the present study—may reveal important differences obscured by quantification numbers alone. Although recent reports have suggested improvements in the known limitations of humoral responses in certain HIS mouse systems (73), these findings have not yet been broadly reproduced, and the extent to which class-switched antibody responses can be reliably generated in current models remains an open question.

Collectively, these data suggest that thymus-supported immune development in HIS mice confers qualitative advantages that become most evident under functional challenge, likely through canonical T cell developmental cues mediated by interactions with human MHC on thymic epithelial cells (74). For vaccine developers, these findings suggest that NeoThy mice may provide a more physiologically relevant environment for evaluating mRNA vaccine-induced T cell responses, repertoire breadth, and potential correlates of protection. Ongoing studies in our laboratory will expand cohort sizes and incorporate antigen-specific functional assays to further validate the NeoThy model as a scalable and translationally relevant research tool.

Importantly, our data did not reveal measurable differences in bulk T cell frequencies that were directly proportional to thymic donor ages, nor did they identify major global disparities between fetal (BLT model) and non-fetal platforms (NeoThy model) beyond differences in T cell expansion kinetics. Future studies incorporating alloreactivity, pathogen challenge models, and longer-term follow-up beyond 36 weeks may provide additional insights. In contrast to the NeoThy model, where thymic donor age can vary widely, fetal BLT mice are generated from tissues within a narrow gestational window, limiting experimental comparisons of donor age. One exception is the propagated BLT (proBLT) model (13), in which HSPCs and thymic tissue from an established BLT mouse are transferred into secondary recipients. In this setting, the kinetics and magnitude of T cell engraftment shift relative to the original BLT animal, suggesting that immune maturation states may evolve during serial *in vivo* propagation. However, the mechanisms underlying these changes and any functional differences between the models remain unclear.

Prior work has suggested that fetal T cells may exhibit distinct regulatory biases (17) associated with the evolutionary pressure to avoid immunological conflict at the maternal-fetal interface (18, 70, 75, 76). Further exploration of developmental programming differences using single-cell transcriptomics and epigenomic profiling represents a logical next step for our studies. Such studies will need to account for evolving legal, regulatory, and funding landscapes surrounding fetal tissue research (14). More broadly, understanding how thymic donor age influences *in vivo* function may have implications extending beyond humanized mouse modeling to clinical thymus transplantation and rejuvenation strategies (77), including applications in congenital athymia (e.g., Rethymic) (31, 78, 79) and immune reconstitution in HIV (80). These systems may also provide experimental leverage for investigating mechanisms of thymic involution and age-associated immune remodeling, although access to adolescent and adult thymus tissue may be more logistically challenging than access to neonatal and pediatric specimens.

Together, these findings indicate that thymic donor age, within the ranges examined here, is unlikely to represent a limiting variable in HIS platform design. Rather, the greater opportunity lies in deliberately leveraging differences among model systems and/or individual donors and in aligning platform choice with the specific scientific question being addressed, thereby unlocking the full translational potential of this important class of HIS models.

## Data Availability

The data presented in the study are deposited in the NCBI BioSample database repository, accession numbers--SAMN61090287, SAMN61090288, SAMN61090289, and SAMN61090290.
