# Supplementary figures and images for: Neonatal and pediatric thymic grafts generate similar human T-cell chimerism in humanized mice
Source: Front Immunol. 2026 Jul 6;17:1852823. doi: 10.3389/fimmu.2026.1852823 (PMC13381199; doi:10.3389/fimmu.2026.1852823)

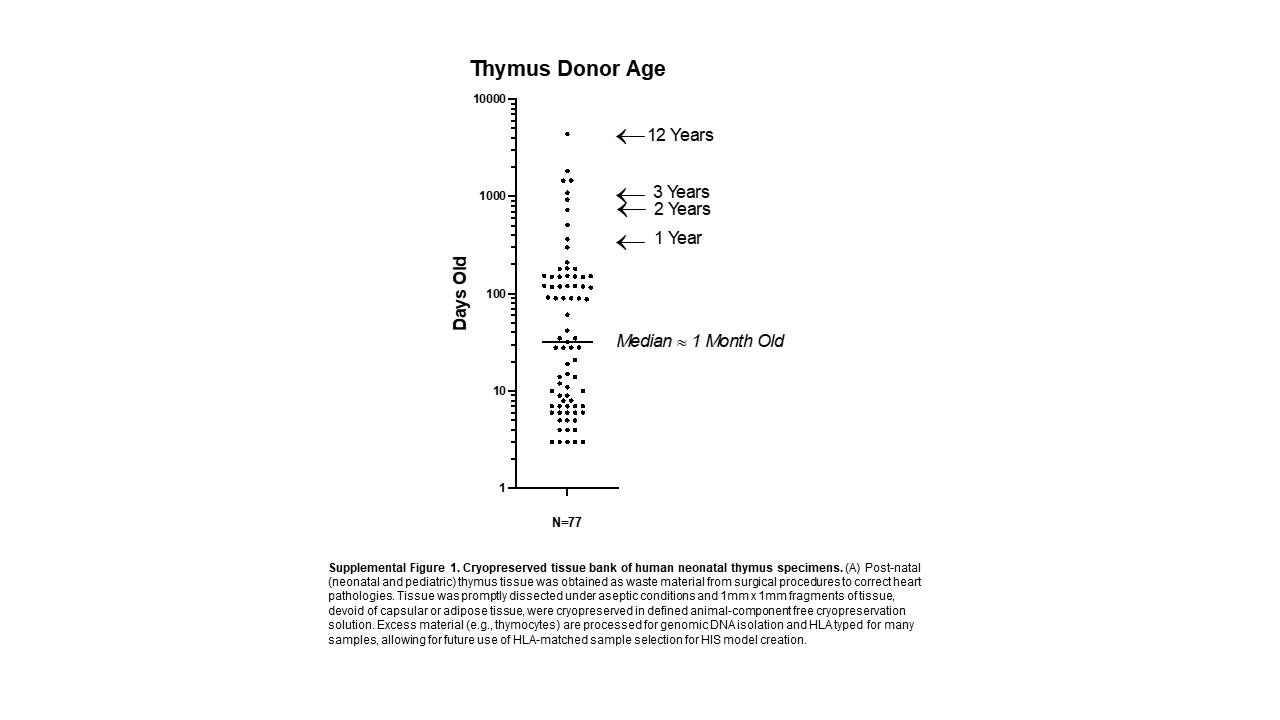

Supplement: Supplementary file 1 [file Image1.jpeg]

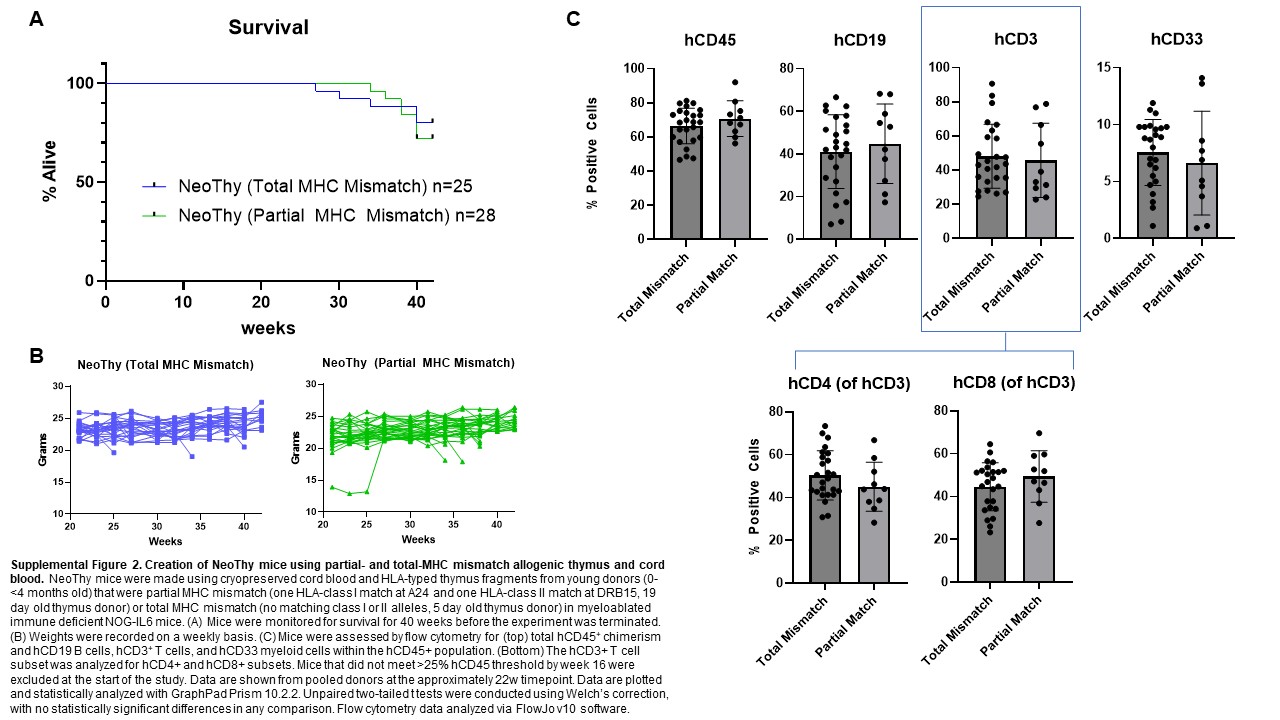

Supplement: Supplementary file 2 [file Image2.jpeg]

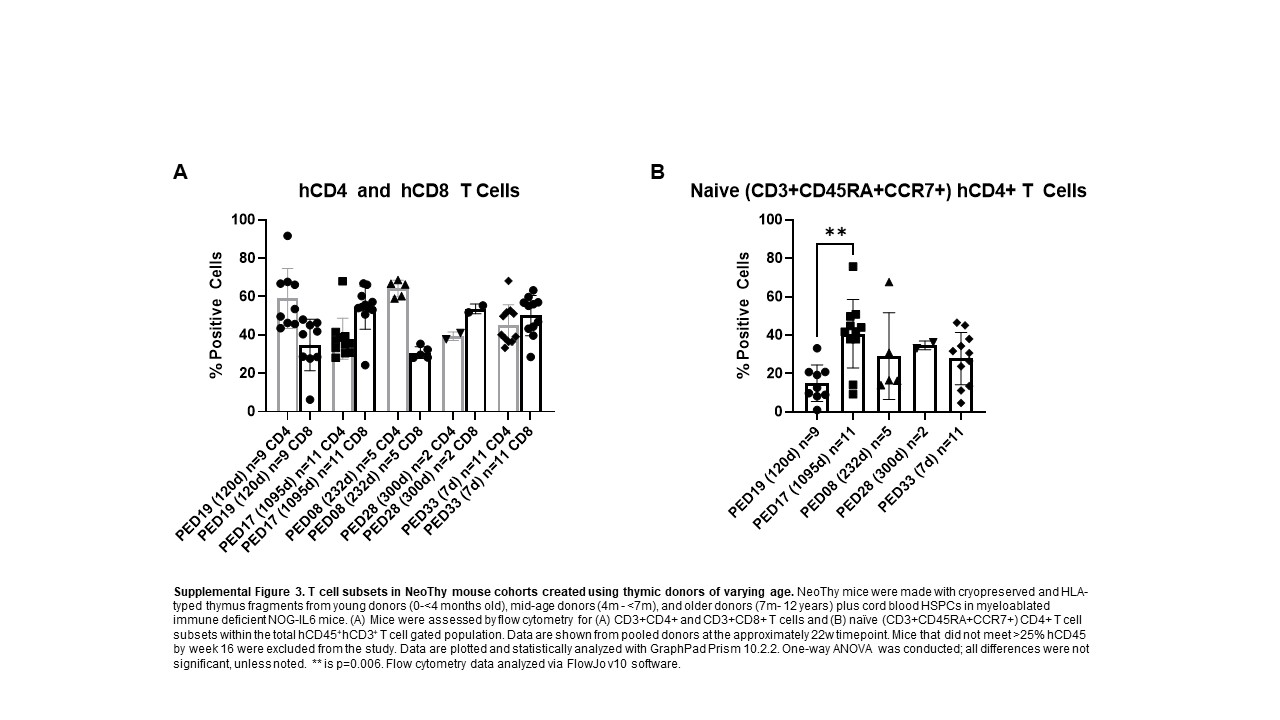

Supplement: Supplementary file 3 [file Image3.jpeg]

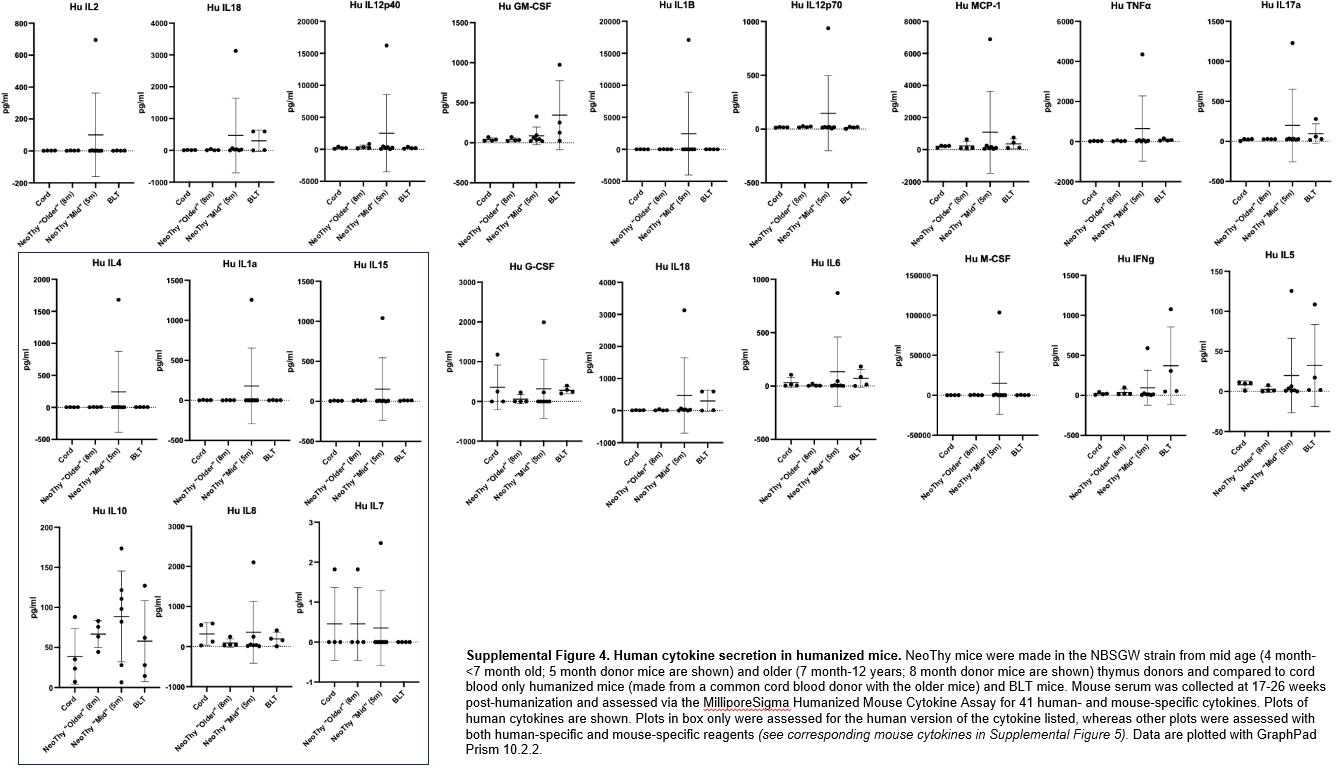

Supplement: Supplementary file 4 [file Image4.png]

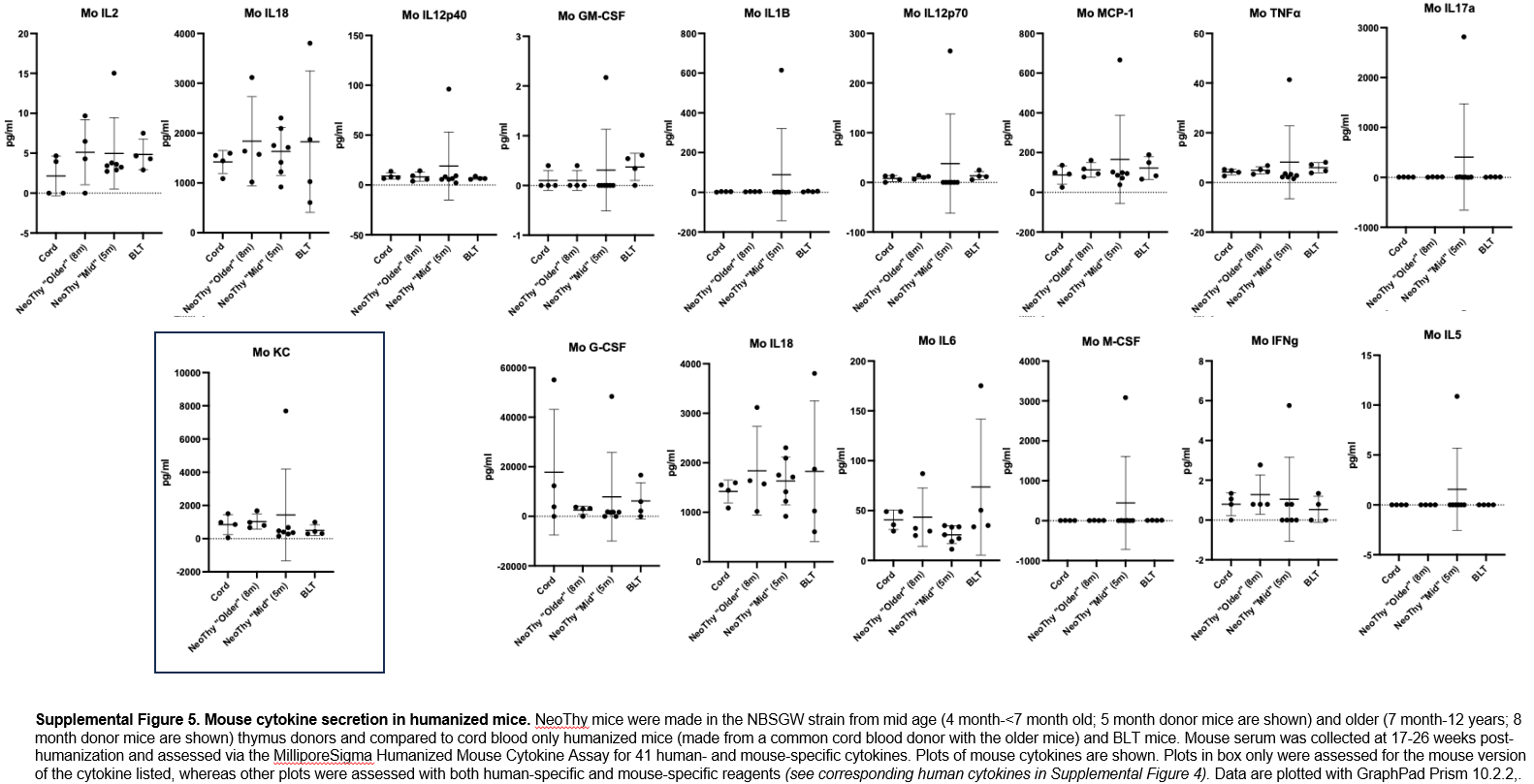

Supplement: Supplementary file 5 [file Image5.png]

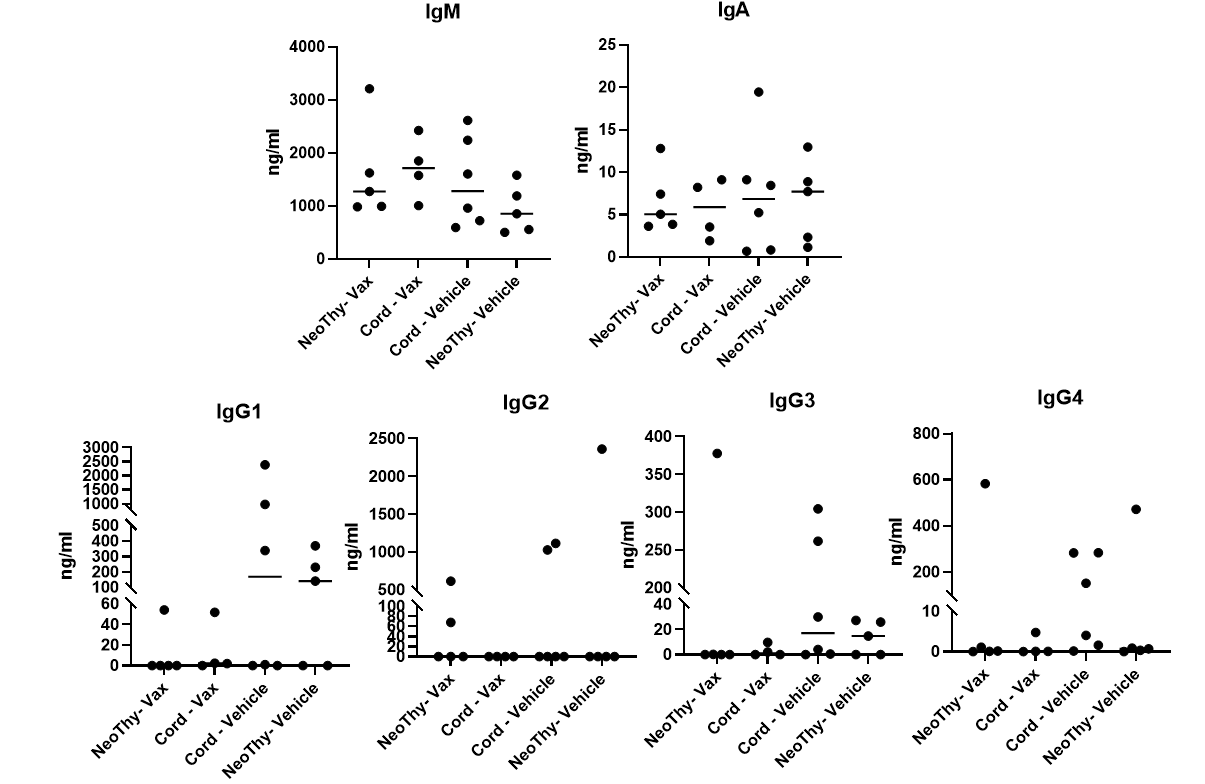

Supplement: Supplementary file 6 [file Image6.png]
